# Adaptive High-Precision 3D Reconstruction of Highly Reflective Mechanical Parts Based on Optimization of Exposure Time and Projection Intensity

**DOI:** 10.3390/jimaging11050149

**Published:** 2025-05-08

**Authors:** Ci He, Rong Lai, Jin Sun, Kazuhiro Izui, Zili Wang, Xiaojian Liu, Shuyou Zhang

**Affiliations:** 1 School of Mechanical Engineering, Yangzhou University, Yangzhou 225127, China; mz120220865@stu.yzu.edu.cn (R.L.); sunjin@yzu.eu.cn (J.S.); 2 School of Mechanical Engineering, Zhejiang University, Hangzhou 310058, China; ziliwang@zju.edu.cn (Z.W.); liuxj@zju.edu.cn (X.L.); zsy@zju.edu.cn (S.Z.); 3 Department of Precision Engineering, Graduate School of Engineering, Kyoto University, Kyoto 615-8540, Japan; izui.kazuhiro.8c@kyoto-u.ac.jp

**Keywords:** 3D reconstruction, highly reflective mechanical parts, multi exposure, image fusion, machine vision

## Abstract

This article is used to reconstruct mechanical parts with highly reflective surfaces. Three-dimensional reconstruction based on Phase Measuring Profilometry (PMP) is a key technology in non-contact optical measurement and is widely applied in the intelligent inspection of mechanical components. Due to the high reflectivity of metallic parts, direct utilization of the captured high-dynamic-range images often results in significant information loss in the oversaturated areas and excessive noise in the dark regions, leading to geometric defects and reduced accuracy in the reconstructed point clouds. Many image-fusion-based solutions have been proposed to solve these problems. However, unknown geometric structures and reflection characteristics of mechanical parts lead to the lack of effective guidance for the design of important imaging parameters. Therefore, an adaptive high-precision 3D reconstruction method of highly reflective mechanical parts based on optimization of exposure time and projection intensity is proposed in this article. The projection intensity is optimized to adapt the captured images to the linear dynamic range of the hardware. Image sequence under the obtained optimal intensities is fused using an integration of Genetic Algorithm and Stochastic Adam optimizer to maximize the image information entropy. Then, histogram-based analysis is employed to segment regions with similar reflective properties and determine the optimal exposure time. Experimental validation was carried out on three sets of typical mechanical components with diverse geometric characteristics and varying complexity. Compared with both non-saturated single-exposure techniques and conventional image fusion methods employing fixed attenuation steps, the proposed method reduced the average whisker range of reconstruction error by 51.18% and 25.09%, and decreased the median error by 42.48% and 25.42%, respectively. These experimental results verified the effectiveness and precision performance of the proposed method.

## 1. Introduction

Three-dimensional profilometry based on structured light techniques has become increasingly prevalent in fields such as advanced manufacturing, quality inspection [1,2,3], intelligent surgery [4], and virtual reality [5], because of its advantages of non-contact operation, full-field measurement, high precision, and efficiency. Phase Measuring Profilometry (PMP) is a widely used frequency encoding method that extracts depth information by analyzing the phase shift resulting from variations in the height of an object’s surface. PMP mitigates the common information loss issue in spatial encoding methods and has advantages in reconstruction efficiency and flexibility compared with temporal encoding techniques.

High-quality, high-precision 3D reconstruction relies on high-quality images, which are, however, significantly influenced by surface reflective properties. There are both highly reflective, polished surfaces and rough, diffuse surfaces in mechanical components, so light observed from a metal surface is obtained after both specular and diffuse reflections. It is difficult to obtain information about all surfaces optimally and simultaneously with a single exposure. We usually increase the exposure to capture details on non-polished surfaces, which results in changes to the encoding information of structured light or exceeding the sensor’s response range. Reconstruction error or information loss would happen in the saturation area. If the exposure is reduced to recover 3D information on bright, polished areas, the signal-to-noise ratio (SNR) significantly drops in dark regions, leading to large decoding errors and reconstruction accuracy loss. This disparity negatively impacts the efficiency and accuracy of PMP-based 3D profilometry using single-frame projected images.

Various solutions have been proposed to enhance the accuracy of PMP-based 3D profilometry considering the multi-reflection characteristics. Major techniques include polarization filter, adjustment of the intensity of projection patterns, multi-exposure-based methods, phase compensation method, deep learning-based method, improved coding method, and color invariant method [6].

One existing method is the use of a polarization filter to extend polarization information from conventional intensity, frequency, and coherence. Salahieh et al. [7] proposed a multi-polarization fringe projection imaging technique to eliminate saturated or low-contrast fringe regions by optimizing combinations of polarization angles and exposure times. Huang et al. [8] proposed a polarization-coded structured light system designed to enhance both efficient 3D reconstruction and polarimetric target detection. By estimating the degree of linear polarization, targets within the scene were effectively distinguished and reconstructed with high efficiency. Xiang et al. [9] introduced a polarization spatial phase-shifting technique using two orthogonally positioned filtered projectors to cast sinusoidal fringe patterns with distinct phase shifts onto the measured metal surfaces. To ensure accurate alignment of the projected patterns, a fringe registration method based on the epipolar geometry between the projectors was also developed. Zhu et al. [10] proposed a polarization-enhanced fringe pattern method to achieve high dynamic range imaging in a single exposure. The degree of linear polarization is precisely calculated by leveraging the polarization properties of reflected light and a fixed-azimuth linear polarizer. They further extended the approach to build a structured light encoding model in Zhu et al. [11], utilizing the superposition of multiple polarization states. This method enables the generation of polarized structured light containing phase information without the need to rotate the polarizer.

To overcome the image saturation problem in 3D reconstruction, methods for adjusting the intensity of projection patterns have been proposed. Li et al. [12] introduced an adaptive fringe pattern projection method to dynamically adjust the projector’s maximum input gray levels based on the local reflectivity of the target surface. In this approach, regions with high reflectivity were illuminated using lower intensity levels to prevent image saturation, while regions with low reflectivity received the highest possible intensity to ensure sufficient intensity modulation for accurate measurement. Li et al. [13] presented an adaptive digital fringe projection technique to calculate the proper intensity for each projector pixel using binary search. The calculation process is simplified compared with previous adaptive techniques because there is no need to obtain the camera response function and homographic mapping between the camera and projector. Chen et al. [14] advanced the fringe projection method to achieve high measurement accuracy. They projected three sets of orthogonal color fringe patterns and a sequence of uniform gray-level patterns with different gray levels onto a measured surface, and captured the deformed patterns by a camera from a different viewpoint. Xu et al. [15] combined adaptive fringe projection with the curve fitting method to compensate for highly reflective surfaces. The optimal light intensity coefficient template of the projection image was calculated to adaptively adjust the projected light intensity based on the pixel mapping and the coefficient template. The 3D reconstruction results were compensated by curve fitting in the horizontal and vertical directions. Zhang et al. [16] projected a small number of uniform grayscale pattern sequences to mark the saturated area and calculate the surface reflection factor and the environment factor of individual pixels. A surface coefficient look-up table was created to calculate the optimal projection intensities of pixels in the saturated regions.

Multi-exposure-based methods improve the quality of the fringe pattern by fusing images acquired at different exposure times. Zhang et al. [17] presented a rapid and fully automatic exposure time determination method by analyzing the texture image acquired by the camera. The reflectivity of the object surface was estimated through a single exposure to determine the global optimal exposure time. Jiang et al. [18] proposed a high dynamic range fringe acquisition to solve the problem caused by a high-reflective surface. They developed a fringe image fusion algorithm to prevent saturation and under-illumination by selecting pixels with the highest fringe modulation intensity from the raw fringe images. Cui et al. [19] introduced a multiple-exposure adaptive selection algorithm. Exposure time nodes are adaptively selected based on the relative irradiance value to cover the highest and lowest gray value of the fringe image. The information entropy theory is introduced in Chen et al. [20] to adaptively optimize the initial exposure value through feature analysis of fringe image entropy. An effective exposure sequence is then generated using the dichotomy method. Zhu et al. [21] proposed an HDR surface 3D reconstruction method based on a shared phase demodulation mechanism and a multi-indicator guided phase fusion strategy. Exposure quality, phase gradient smoothness, and pixel effectiveness were included in a phase sequence fusion model to obtain an optimum phase map for final 3D reconstruction.

Phase compensation methods apply post-processing techniques to images acquired by cameras to recover fringe pattern information that is distorted or lost due to reflective interference. Hu et al. [22] proposed a dynamic phase retrieval algorithm based on unsaturated frame data. By defining a saturation coefficient K to analyze error characteristics and validating the approach through simulations, the method effectively addresses phase distortion issues arising in highly reflective surfaces or scenarios with limited dynamic range. Budianto et al. [23] developed a fringe projection profilometry restoration technique based on geometry-guided iterative regularization. This method employs a Gaussian Mixture Model to detect specular regions, generates an initial fringe structure via geometric sketching, and iteratively refines the result using a dual-tree complex wavelet transform. Ren et al. [24] introduced a specular reflection separation approach based on a global color-line constraint. The method estimates illumination chromaticity by analyzing intersection points of color lines in normalized RGB space, clusters specular-invariant variables, and separates specular components at the pixel level based on their distance to the estimated illumination chromaticity. Chen et al. [25] proposed a high-frequency averaged phase compensation method guided by an optimal frequency strategy. By reducing the number of projected images and incorporating both high-frequency phase compensation and model-driven optimal frequency selection, the method significantly suppresses phase errors caused by gamma nonlinearity.

In a deep learning-based method, Zhang et al. [26] designed a specialized convolutional neural network that takes high dynamic range (HDR) fringe patterns with three-step phase shifting as input, enabling accurate extraction of phase information in both low signal-to-noise ratio (SNR) and HDR scenes. Liu et al. [27] proposed a Skip Pyramid Context Aggregation Network (SP-CAN) to enhance fringe images captured synchronously by a single-exposure camera, while precisely preserving encoded phase details near edges and corners. Shen et al. [28] employed an improved UNet-based deep neural network to establish a “many-to-one” mapping, utilizing π-phase-shifted binary fringes to acquire more saturated fringe information, thereby enabling fast and accurate retrieval of wrapped phase maps for HDR objects. Xi et al. [29] developed an encoder–decoder network guided by reflection priors to restore defective fringe patterns caused by highly reflective surfaces. This method transforms distorted fringes into ideal patterns with uniform grayscale distribution, effectively eliminating reflective artifacts and recovering the missing phase information.

Improved encoding methods have been developed based on advanced projection coding strategies. Song et al. [30] proposed a structured light approach based on fringe edges, which integrates gray code with a positive-negative fringe pattern encoding scheme. An enhanced zero-crossing edge detector is employed to achieve subpixel-level edge localization. Tang et al. [31] introduced a micro-phase measurement profilometry technique that improves both the accuracy and efficiency of shape acquisition while demonstrating strong robustness against global illumination variations. Feng et al. [32] presented a fast 3D measurement method combining dual-camera fringe projection with digital speckle image fusion. By leveraging trifocal tensor constraints to correct phase errors in highly reflective regions and employing a three-step phase-shifting algorithm along with subpixel matching, the method enables efficient and high-precision 3D reconstruction of reflective surfaces in dynamic scenes using only four projected patterns. Zhao et al. [33] developed an adaptive checkerboard high-frequency projection technique, which integrates high-frequency encoded patterns with complementary projection and dynamically adjusts the projection intensity to suppress image saturation caused by specular highlights, thereby significantly enhancing the measurement accuracy and point cloud completeness for highly reflective surfaces.

Color-based specular highlight removal methods are primarily founded on the dichromatic reflection model proposed by Shafer [34], which separates specular and diffuse reflection components by analyzing the color distribution of pixels in RGB images, thereby facilitating the estimation of surface normals for each pixel. Benveniste et al. [35] developed a structured light range scanner based on color invariance, employing binary, ternary, and quaternary encoding schemes to robustly scan both glossy and matte objects under ambient lighting conditions. Xu et al. [36] proposed an adaptive fringe projection framework based on the dichromatic reflection model, which precisely identifies specular regions by decomposing specular and diffuse components. The method suppresses specular reflections by optimizing projection intensity using a power function and restores missing information through pixel inpainting techniques. Feng et al. [37] presented a specular highlight removal method for light field images that combines the dichromatic reflection model (DRM) with exemplar-based patch filling. Specular pixels are first classified using a Gaussian Mixture Model clustering and depth-based segmentation. Non-saturated highlights are removed using a DRM confidence strategy, while saturated highlights are addressed using an exemplar patch matching algorithm that integrates gradient and color difference constraints.

However, limits are exhibited when using the mentioned approaches to measure mechanical components with unknown geometries and highly reflective surfaces. While incorporating a polarization device can effectively suppress high-reflection surfaces, it also reduces the signal-to-noise ratio (SNR) for low-reflection surfaces. Also, precise optical equipment and high environmental requirements are often necessary, which increases hardware costs and makes it difficult to apply to mechanical industrial inspections in complex environments. Multiple exposure techniques can enhance the SNR by integrating images captured at varying exposure times, but they traditionally rely on the empirical knowledge of experimentalists, which may limit the generalizability and reduce reconstruction accuracy. Phase compensation methods are not suitable for high-precision measurements, as they involve complex iterative and growing processes for edge image restoration. Deep learning-based methods offer higher efficiency but require the establishment of extensive datasets for training. Improved encoding methods achieve high reconstruction accuracy, but they necessitate more complex phase unwrapping techniques. Color invariance methods can produce good reconstruction results, but they are influenced by the object’s surface color and texture. Further, adjusting the projection intensity can enhance the SNR, but it is subject to the influence of pixel mapping errors and grayscale range limitations between the camera and the projector.

Therefore, adjusting a single imaging parameter cannot effectively adapt to the actual part geometry and surface reflective properties. Existing methods lack effective guidance on the design of these important imaging parameters.

To address this problem, an adaptive high-precision 3D reconstruction of highly reflective mechanical parts based on optimization of exposure time and projection intensity is proposed in this paper. The projection intensity is optimized based on the linear response range of the hardware to generate an image sequence, which is further fused through an optimization process based on Genetic Algorithm and stochastic Adam optimizer. Region segmentation is conducted to adapt the exposure time to surface reflective properties based on histogram analysis.

This paper is organized as follows: Section 2 briefs the basic theory of PMP-based 3D reconstruction. The proposed image fusion based on projection intensity optimization is displayed in Section 3. Histogram-based analysis of the optimal exposure is demonstrated in Section 4. In Section 5, the advantage of the proposed method is shown by three sets of examples. Finally, conclusions and future research are given in Section 6.

## 2. Basic Theory of PMP-Based 3D Reconstruction

**Figure 1:**
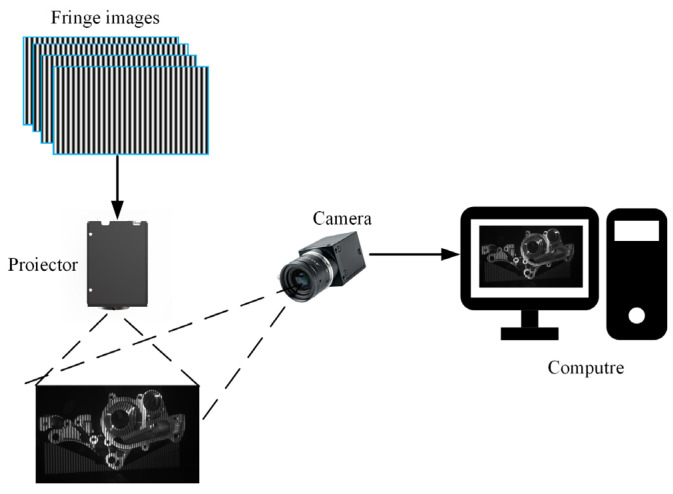
The monocular structured light measurement based on PMP.

The configuration of PMP-based 3D measurement system implemented a monocular structured light measurement architecture, as shown in Figure 1.

Ideal sinusoidal fringe patterns generated by a computer are projected onto the measured object’s surface through a Digital Light Processing (DLP) projector. Then the deformed fringe patterns modulated by the object’s surface geometry are synchronously captured by a CCD camera.

The computer-generated sinusoidal fringe patterns could be expressed as:

(1) $$I_{i}\left(x,y\right)=I_{a}\left(x,y\right)+I_{b}\left(x,y\right)\cos\left[\varphi \left(x,y\right)+\delta_{i}\right],$$

where $I_{a}\left(x,y\right)$ represents the average light intensity, $I_{b}\left(x,y\right)$ is the modulated light intensity, $\varphi \left(x,y\right)$ is the wrapped phase to be solved, $\delta_{i}$ is the phase shift amount of the i-th image, i=1,2,3,…,N, $\delta_{i}=2\pi i/N$, and N denotes the total number of phase-shifting steps. The wrapped phase $\varphi \left(x,y\right)$ could be determined as follows:

(2) $$\varphi \left(x,y\right)=-\arctan\left[\frac{\sum_{i=0}^{N-1}I_{i}\left(x,y\right)\sin\left(\delta_{i}\right)}{\sum_{i=0}^{N-1}I_{i}\left(x,y\right)\cos\left(\delta_{i}\right)}\right],$$

After obtaining the wrapped phase, the absolute phase containing height information could be retrieved through the heterodyne multi-frequency phase-shifting approach [38]. System calibration is then conducted following Song et al.’s method [39], in which the DLP projector is treated as an inverse camera. The calibrated parameters contribute to geometric reconstruction based on triangulation principles.

## 3. Image Fusion Based on Projection Intensity Optimization

To accurately recover information in saturated regions, existing studies typically employ a fixed intensity attenuation step size to ensure that the saturated regions are no longer saturated. However, this method is limited by the adaptability and often requires manual intervention when dealing with various surfaces with diverse geometric and material properties.

Therefore, an adaptive method for image fusion based on projection intensity optimization is proposed in this section. The gist of this method is to effectively optimize the projection intensity to ensure that the acquired images in each segmented region remain within the mid-to-high linear response grayscale range, which is typically determined by the camera hardware. As the projection intensity decreases, the upper limit of the best range $R_{u}$ is utilized to measure the change in the number of pixels in the linear response range: binarize both the initial image $I_{0}^{c}(I_{0}^{p})$ and the projected images $\left\{I_{i}^{c}\left(I_{i}^{p}\right)|i=1,2,\ldots ,N\right\}$ captured by the camera, in order to extract the difference regions $D_{i}^{c}$ with varying grayscale values:

(3) $$\left\{\begin{array}{c} U_{i}=\sum_{j=1}^{m}\left(D_{0}^{c(j)}-D_{i}^{c(j)}\right)\\ D_{i}^{c(j)}=\left\{\begin{array}{c} 1,\;I_{i}^{c(j)}\left(I_{i}^{p}\right)\geq R_{u}\\ 0,\;otherwise \end{array}\right. \end{array}\right.,i=0,1,\ldots ,N,$$

where $I_{0}^{p}$ represents the initial projection intensity, $I_{i}^{p}$ is the projection intensity at iteration i, $D_{i}^{c}$ is the difference mask when the projection intensity varies. From Equation (3), it is evident that at iteration i, the difference projection intensity $I_{i}^{p}$ are projected onto the workpiece to compute the difference region $D_{i}^{c}$. Since different segmented regions possess distinct optimal projection intensities, they are processed separately and parallelly in subsequent calculations.

To accurately recover information in saturated regions, the projection intensity is typically reduced gradually from 255. When the grayscale range of the difference region aligns with the camera’s linear response and high SNR capture interval $\left[R_{l},R_{u}\right]$, the projection intensity is considered to achieve its optimal, which should be recognized and encouraged quantitatively in the optimization. Therefore, an objective function is constructed to measure the deviation between the obtained grayscale interval and the optimal range:

(4) $$f_{D}^{\left(j\right)}\left(I_{i}^{p}\right)=\min\left(\max\left(D_{i}^{c(j)}⨀U_{i}\right)-{Int}_{u}^{c},{Int}_{l}^{c}-\min\left(D_{i}^{c(j)}⨀U_{i}\right)\right),$$

where ⨀ indicates the convolution operator. When the obtained dynamic range of the difference image exceeds the upper limit of the optimal range or falls below the lower limit, the objective function $f_{D}\left(I_{i}^{p}\right)$ returns a large positive value. On the contrary, the objective function would show a negative value if the dynamic range $D_{i}^{c}$ falls within the optimal range. Therefore, minimizing this objective function tends to keep the captured image in conformation to the hardware’s optimal range.

Then, a simulated annealing algorithm is implemented in this paper to optimize the objective function and achieve the optimal projection intensity. The main steps are as follows:

(1) Calibrate the hardware to obtain the optimal range $\left[R_{l},R_{u}\right]$. Set the initial projection intensity $I_{0}^{p}$ to the maximum value of 255. Acquire the baseline image $I_{0}^{c(j)}$ of the j-th cluster with the camera, and perform binarization to get $D_{0}^{c(j)}$. Initialize i=0.
(2) Let i=i+1. Calculate the i-th projection intensity using an attenuation step S, and conduct a physical experiment to observe the captured image of the j-th cluster $I_{i}^{c(j)}\left(I_{0}^{p}-iS\right)$. Obtain the corresponding difference image $D_{i}^{c(j)}$ and the value of objective function $f_{D}^{\left(j\right)}\left(I_{i}^{p}\right)$ in each cluster based on Equations (3) and (4).
(3) Randomly generate a new projection intensity ${\hat{I}}_{i}^{p}$ in the neighborhood of variable $I_{i}^{p}$. Observe the captured image $f_{D}\left({\hat{I}}_{i}^{p}\right)$, and evaluate the objective function in each cluster $f_{D}^{\left(j\right)}\left({\hat{I}}_{i}^{p}\right)$ to compare with the current one. If the new value is superior, accept the new projection intensity. Otherwise, accept it with a probability $P_{i}$, in which $T_{0}$ denotes the initial temperature and α is the cooling speed.
  (5) $$P_{i}=\exp\left(-\frac{f_{D}^{\left(j\right)}\left({\hat{I}}_{i}^{p}\right)-f_{D}^{\left(j\right)}\left(I_{i}^{p}\right)}{T_{0}\alpha^{t}}\right),$$
(4) If the temperature has dropped below a threshold, or the optimal solution has not been updated in multiple consecutive iterations, the iteration process is terminated. Go to step (5). If the iteration has not been terminated and $f_{D}^{\left(j\right)}\left(I_{i}^{p}\right)<0$, store the newly acquired image $f_{D}^{\left(j\right)}\left(I_{i}^{p}\right)$, go back to step (2) and substitute the baseline image $I_{0}^{c}$ with $f_{D}^{\left(j\right)}\left(I_{i}^{p}\right)$.
(5) Consider current $I_{i}^{p}$ as the optimal projection intensity. Output all the stored images as an image sequence $\left\{I_{i}^{s},i=1,2,\ldots ,L\right\}$. Terminate the algorithm when the optimization processes of all regions have converged.

Then, an image fusion method is proposed to recover information in saturated regions based on the complementarity of multi-source image sequence. Image information entropy is utilized to quantitatively evaluate the informational richness of fused images, serving as a metric to assess the complexity of grayscale distribution statistics, as shown in Equation (6).

(6) $$H\left(I_{i}^{s}\right)=-\sum_{j=1}^{n_{i}}p_{i,j}\log\left(p_{i,j}\right),i=1,2,\ldots ,L,$$

where $p_{i,j}$ is the probability of grayscale j in the i-th fused image, and $n_{i}$ is the number of grayscale levels. Information entropy $H(I_{i}^{s})$ quantifies the disorder and variability in pixel intensity distributions. Oversaturated areas and dark areas show low information entropy because pixels’ grayscales are clustered in high-intensity or low-intensity ranges, while higher entropy reflects greater complexity in grayscale distributions and enhanced preservation of fine image details.

The core challenge of image fusion lies in optimizing the retained region from each image in the sequence to ensure that the fused image achieves maximal information entropy. Assume the image sequence is arranged in ascending order of its mean grayscale values. A threshold $T_{i}^{f}$ is applied to the segmentation of the i-th grayscale image, in which higher-intensity regions are consistently prioritized for retention to enhance the SNR of the fused output. The segmented region marked by $D_{i}^{s}$ is retained in the final fused image. The newly acquired region marked by $D_{i}^{s}$ at iteration i is consistently preserved during subsequent image fusion operations.

(7) $$D_{i}^{s}\left(x,y\right)=\left\{\begin{array}{c} 1,I_{i}^{s}\left(x,y\right)\geq T_{i}^{f}\;and\;D_{i-1}^{s}\left(x,y\right)=0\\ 0,otherwise \end{array}\right.,$$

Therefore, the image fusion process from the obtained image sequence $\left\{I_{i}^{s},i=1,2,\ldots ,L\right\},$ can be formulated in:

(8) $$I_{L}^{f}=\left(D_{1}^{s}⨀I_{1}^{s}\right)\cup \left(D_{2}^{s}⨀I_{2}^{s}\right)\cup \ldots \cup \left(D_{L}^{s}⨀I_{L}^{s}\right),$$

Then, the L-dimensional segmentation thresholds $\left\{T_{i}^{f},i=1,2,\ldots ,L\right\}$ are obtained through a recursive process of Genetic Algorithm (GA)-based [40] global optimization and local fine-tuning based on stochastic Adaptive Moment Estimation (S-Adam). GA explores optimal solutions by simulating evolutionary processes. GA approaches are able to escape local optima through their stochastic operations, while requiring substantial computational resources and relatively slow convergence rates, especially for multi-dimensional problems. Adam [41] shows superior converging performance in high-dimensional parameter spaces, while remaining susceptible to local optima entrapment and sensitive to the initial value. To overcome these limitations, a hybrid optimization framework is proposed in this paper in which the elite solution from each GA generation serves as the initial parameter set for Adam-based local refinement. In addition, the mini-batch gradient method is used to achieve a balance between computational efficiency and convergence stability.

The chromosome representing segmentation thresholds $\left\{T_{i}^{f},i=1,2,\ldots ,L\right\}$ is encoded using a binary scheme, where each individual consists of L chromosomes. The algorithm begins by generating an initial population, and the fitness function is constructed based on image information entropy $H\left(I_{L}^{s}\right)$. The top K individuals with the highest fitness values are fine-tuned through the Adam optimizer and subsequently reintroduced into the population. The remaining individuals undergo selection using the roulette wheel method. As the population iteratively evolves, new individuals are generated through crossover and mutation operations. The crossover operation involves exchanging chromosomes between two distinct parent individuals, while the mutation operation randomly alters selected genes.

To mitigate premature convergence to local optima, the variance of population fitness is employed as a convergence metric. The effectiveness of crossover and mutation operations diminishes as evolutionary iterations progress and individual fitness values gradually converge. To address this challenge, an adaptive adjustment mechanism is proposed for modifying the crossover probability $p_{i}^{c}$ and mutation probability $p_{i}^{m}$.

(9) $$p_{i}^{c}=\left\{\begin{array}{c} p_{i-1}^{c}-\frac{p_{i-1}^{c}}{std_{i}-std_{i-1}},std_{i}>std_{i-1}\\ p_{i-1}^{c}+\frac{p_{i-1}^{c}}{std_{i-1}-std_{i}},std_{i}<std_{i-1} \end{array}\right.,i\geq 2,$$

(10) $$p_{i}^{m}=\left\{\begin{array}{c} p_{i-1}^{m}-\frac{p_{i-1}^{m}}{std_{i}-std_{i-1}},std_{i}>std_{i-1}\\ p_{i-1}^{m}+\frac{p_{i-1}^{m}}{std_{i-1}-std_{i}},std_{i}<std_{i-1} \end{array}\right.,i\geq 2,$$

where $std_{i}$ is the variance of population fitness at iteration i. Probabilities $p_{i}^{c}$ and $p_{i}^{m}$ are dynamically tuned according to the convergence state to maintain the exploratory capability.

Adam is then implemented to fine tune the top individuals. An independent learning rate is employed for each parameter, and is adjusted based on the first and second moment estimation of the gradient:

(11) $$\left\{\begin{array}{cc} m_{t} & =\beta_{1}m_{t-1}+\left(1-\beta_{1}\right){\hat{g}}_{t}\\ v_{t} & =\beta_{2}v_{t-1}+\left(1-\beta_{2}\right){\hat{g}}_{t}^{2}\\ \theta_{t+1} & =\theta_{t}-\frac{\eta_{1}m_{t}}{\left(1-\beta_{1}^{t}\right)\sqrt{t\left(\frac{v_{t}}{1-\beta_{2}^{t}}+\varepsilon \right)}} \end{array}\right.,$$

where $m_{t}$ represents the first moment of gradient $g_{t}$ at iteration t, $v_{t}$ is the second moment, $\beta_{1}$ and $\beta_{2}$ are decaying speed hyperparameters satisfying $\beta_{1},\beta_{2}\in \left[0,1\right]$, $\beta_{1}<\beta_{2}$, $\theta_{t}=\left(T_{1}^{f},T_{2}^{f},\ldots ,T_{L}^{f}\right)$ is the vector of parameters, $\eta_{t}=\eta_{1}/\sqrt{t}$ is a decaying learning rate, ε is an artificial small value to avoid vanishing gradient problem. The gradient $g_{t}$ here is estimated by the difference of adjacent $T_{i}^{f}$ in dimension i. In order to reduce the amount of gradient calculation in each iteration and increase the stability of convergence, the mini-batch method is implemented:

(12) $${\hat{g}}_{t}=\frac{1}{B}\sum_{i=1}^{B}\frac{H\left(\theta_{t,i}+\Delta I\right)-H\left(\theta_{t,i}\right)}{\Delta I},$$

where B is the batch size, B<L, ΔI is a small increase.

After the algorithm converges, the optimal segmentation thresholds are substituted into Equations (7) and (8) to generate the fused image for subsequent calculation of the optimal exposure time.

## 4. Histogram-Based Analysis of the Optimal Exposure

**Figure 2:**
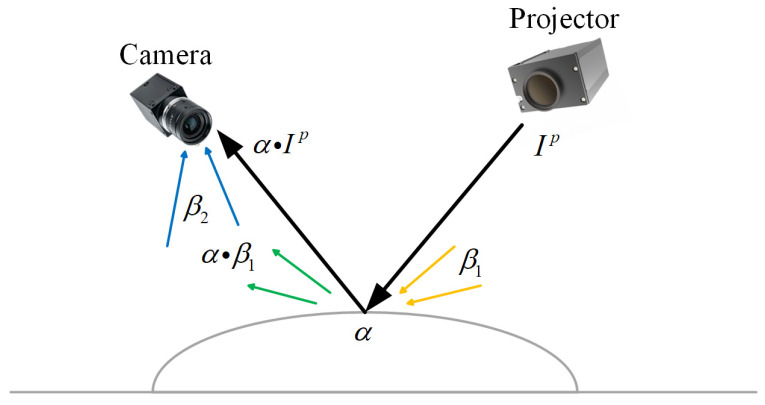
Illustration for PMP-based 3D reconstruction of mechanical parts.

For mechanical parts with high reflectivity, the exposure time significantly influences fringe pattern quality and consequently impacts 3D reconstruction accuracy when using structured light measurement systems. As shown in Figure 2, the optimal exposure time should be determined adaptively by the surface reflectivity of the measured part and the ambient illumination, which is considered constant in this paper. Basically, shorter exposure times should be employed to prevent overexposure in high reflective regions, while surfaces with lower reflectivity require extended exposure durations to enhance the SNR of captured surfaces. Although this principle is widely recognized, the determination of specific exposure time still predominantly relies on empirical judgment. Such manual approaches lack adaptability under dynamic scenarios of the mechanical parts and ambient lighting, which results in a compromised reconstruction accuracy. Therefore, a histogram-based analysis method is introduced to obtain region-specific optimal exposure times.

When projecting uniform and high-intensity white light, the intensity distribution of the images captured by the camera can be expressed as:

(13) $$I^{c}=st\alpha I^{P}+\alpha \beta_{1}+\beta_{2},$$

where s and t represent the sensitivity and exposure of the camera, respectively, α is derived from geometric and material properties, $I^{P}$ is the intensity of projected light, a term $\alpha \beta_{1}$ denotes ambient light reflected by the measured object, and $\beta_{2}$ is ambient light directly entering the camera. Assume the relationship between image intensity and scene radiance is linear, which means the camera sensitivity s is considered constant. Consider a segmented pixel cluster marked by j in m clusters, Equation (13) can be discretized into a partition expression:

(14) $$I^{c\left(j\right)}=st^{\left(j\right)}\alpha^{\left(j\right)}I^{p\left(j\right)}+\alpha^{\left(j\right)}\beta_{1}^{\left(j\right)}+\beta_{2}^{\left(j\right)},j=1,2,\ldots ,m,$$

A radiometric compensation strategy is employed to process high reflective surfaces, in which the projection intensity $I^{P\left(k\right)}$ is systematically varied while maintaining the initial exposure time t. A sequence of intensity images $I_{seq}^{c}$ is obtained by the camera, where pixel saturation in some overexposed regions is progressively alleviated across different intensity levels $\left\{I_{1}^{p},I_{2}^{p},\ldots ,I_{n}^{p}\right\}$. These multi-intensity segmented pixel regions would be subsequently fused into a composite image using the method detailed in Section 3. Assume n distinct projection intensities are applied to generate a sequence of images $\left\{I_{1}^{c},I_{2}^{c},\ldots ,I_{n}^{c}\right\}$, ensuring a comprehensive coverage of both specular and diffuse reflection characteristics:

(15) $$I_{i}^{c}=st\alpha I_{i}^{p}+\alpha \beta_{1}+\beta_{2},i=1,2,\ldots ,n,$$

Therefore, the image fusion process based on spatially and temporally discrete sampling can be formulated based on an integration of Equations (14) and (15):

(16) $$I^{f}=\sum_{i=1}^{n}\sum_{j=1}^{m}\lambda_{ij}\left(st^{\left(j\right)}\alpha^{\left(j\right)}I_{i}^{p\left(j\right)}+\alpha^{\left(j\right)}\beta_{1}^{\left(j\right)}+\beta_{2}^{\left(j\right)}\right),$$

where $\lambda_{ij}$ represents the region-adaptive binary weighting function to be obtained in this section, which dynamically prioritizes unsaturated pixel values across temporal and spatial domains. Consider a specific pixel cluster j, if the critical oversaturation intensity is defined as $I^{os}$, the oversaturation threshold exposure time in region j is given by:

(17) $$t_{os}^{\left(j\right)}=\frac{I^{os}-\alpha^{\left(j\right)}\beta_{1}^{\left(j\right)}-\beta_{2}^{\left(j\right)}}{s\alpha^{\left(j\right)}\sum_{i=1}^{n}\lambda_{ij}\left(I_{i}^{p\left(j\right)}\right)},$$

To ensure high SNR in the sampled area and avoid saturation of the camera sensor, the critical oversaturation intensity is usually defined as a constant value [42]. Then, the obtained pixel cluster in region j is:

(18) $$I^{c\left(j\right)}=\alpha^{\left(j\right)}\left(\beta_{1}^{\left(j\right)}-\frac{\beta_{1}^{\left(j\right)}t^{\left(j\right)}}{t_{os}^{\left(j\right)}}\right)+\frac{I^{os}-\beta_{2}^{\left(j\right)}}{t_{os}^{\left(j\right)}}t^{\left(j\right)}+\beta_{2}^{\left(j\right)},$$

**Figure 3:**
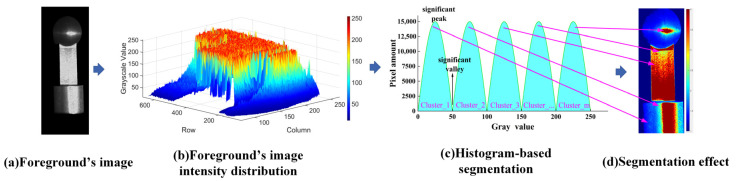
Illustration of histogram-based segmentation.

Parameters $\beta_{1}^{\left(j\right)}$ and $\beta_{2}^{\left(j\right)}$ for a given region, which are related to ambient illumination, pixel positions, materials, roughness, and other intrinsic properties, remain constant during a continuous measurement session. $t_{os}^{\left(j\right)}$ is an unknown constant to be determined. Therefore, as derived from Equation (18), the intensity of a specific region in the acquired image is linearly related to the reflectivity, which further indicates that variations in regional intensity directly reflect changes in surface reflectivity. As shown in Figure 3, pixels with distinct intensity levels can be clustered based on histogram analysis of the foreground’s image from images without highly saturated regions, resulting in an identification and segmentation of regions with statistically homogeneous intra-class reflectivity.

The method for obtaining the foreground image is derived from the literature [43], and is described in detail as follows. For a grayscale image I, it is composed of a foreground image F, a background image B, and the foreground opacity $\alpha_{i}$ at each pixel:

(19) $$I_{i}=\alpha_{i}F_{i}+\left(1-\alpha_{i}\right)B_{i},$$

Assuming that the foreground F and background B are approximately constant within a small window around each pixel, this assumption allows the opacity α to be expressed as a linear function of the image I:

(20) $$\alpha_{i}\approx aI_{i}+b,\;\forall i\in \omega ,$$

where $a=1/\left(F-B\right)$, $b=-B/\left(F-B\right)$, and ω is a small image window.

To solve for α, a and b, a cost function is constructed as follows:

(21) $$J(\alpha ,a,b)=\sum_{j\in I}\left(\sum_{i\in \omega_{j}}{\left(\alpha_{i}-\alpha_{j}I_{i}-b_{i}\right)}^{2}+\epsilon a_{j}^{2}\right),$$

where $\omega_{j}$ is a small window around pixel j.

By minimizing J(α,a,b), a quadratic cost function solely with respect to α is obtained:

(22) $$J(\alpha )=\alpha^{T}L\alpha ,$$

where L is the

(23) $$L_{i,j}=\sum_{k|\left(i,j\right)\in \omega_{k}}\left(\delta_{ij}-\frac{1}{\left|\omega_{k}\right|}\left(1+\frac{\left(I_{i}-\mu_{k}\right)\left(I_{j}-\mu_{k}\right)}{\frac{\epsilon }{\left|\omega_{k}\right|}+\sigma_{k}^{2}}\right)\right),$$

where $\mu_{k}$ and $\sigma_{k}^{2}$ are the mean and variance of the intensities in the window $\omega_{k}$ around k, and $\left|\omega_{k}\right|$ is the number of pixels in this window.

Constraints are added, and the resulting sparse linear system is then solved:

(24) $$(L+\lambda D_{S})\alpha =\lambda D_{S}b_{S},$$

where $D_{S}$ is a diagonal, $b_{S}$ is the constraint vector, and λ is a large constant. The alpha matte α is solved using a sparse linear solver.

Given $I_{i}=\alpha_{i}F_{i}+\left(1-\alpha_{i}\right)B_{i}$, where $F_{i}$ and $B_{i}$ are unknown, the foreground F and background B can be estimated by minimizing an objective function with smoothness priors:

(25) $$\underset{F,B}{min}\sum_{i}{\left(\alpha_{i}F_{i}+\left(1-\alpha_{i}\right)B_{i}-I_{i}\right)}^{2}+\lambda \left(∥\nabla F_{i}{∥}^{2}+∥\nabla B_{i}{∥}^{2}\right),$$

where $\nabla F_{i}$ and $\nabla B_{i}$ denote the image gradients of the foreground and background.

The clustering-based segmentation is performed using the K-means++ algorithm proposed by Arthur et al. [44], with the following steps:

(1) Randomly and uniformly select one point from the dataset χ as the first initial center $c_{1}$.
(2) For each data point x∈χ, compute the distance $D\left(x\right)$ to the nearest already selected center:
  (26) $$D\left(x\right)=\underset{c\in \left\{c_{1},\ldots ,c_{i-1}\right\}}{min}∥x-c{∥}^{2},$$

Select the next center $c_{i}$ from the dataset with probability proportional to ${D\left(x\right)}^{2}$:

(27) $$P(x)=\frac{D(x{)}^{2}}{\sum_{x\in \chi }D(x{)}^{2}},$$

A new point is then randomly selected as $c_{i}$ according to this probability distribution.

(3) Assign each data point to the cluster $C_{i}$ associated with the nearest center $c_{i}$:

(28) $$C_{i}=\left\{x\in \chi |∥x-c_{i}∥\leq ∥x-c_{j}∥,\;\forall j\neq i\right\},$$

where $C_{i}$ denotes the set of points assigned to center $c_{i}$.

(4) Recompute the center of each cluster as the mean of all points within the cluster:

(29) $$c_{i}=\frac{1}{\left|C_{i}\right|}\sum_{x\in C_{i}}x,$$

(5) Iterate until convergence by repeating steps (3) and (4), either until the set of centers no longer changes or the maximum number of iterations is reached.
(6) To ensure coverage of the exposure time for each class, the data point corresponding to the right boundary of each cluster is selected as the segmentation threshold. This data point should satisfy the following condition:

(30) $$\left\{\begin{array}{c} f\left(x_{i+1}\right)-f\left(x_{i}\right)>0\\ f\left(x_{i}\right)-f\left(x_{i-1}\right)<0 \end{array}\right.,$$

Through the aforementioned steps, a relatively accurate segmentation threshold can be obtained.

Following the spatial segmentation, the optimal exposure time is determined for each partitioned region. Evidently, regions with distinct reflectivity require different optimal exposure times. To maximize the SNR of captured fringes, the ideal exposure time should correspond to the critical threshold that prevents saturation in all pixels within the region. Since longer exposure durations increase pixel saturation, the goal is to maximize $t_{os}^{\left(j\right)}$ while ensuring no saturation occurs:

(31) $$t_{opt}^{\left(j\right)}=\max\left(t_{os}^{\left(j\right)}\right)=max\left(\frac{I^{os}-\alpha^{\left(i\right)}\beta_{1}^{\left(j\right)}-\beta_{2}^{\left(j\right)}}{\frac{\delta I^{c(j)}}{\delta t^{\left(j\right)}}}\right),$$

Obviously, the optimal exposure time $t_{opt}^{\left(j\right)}$ is obtained when the term $\delta I^{c\left(j\right)}/\delta t^{\left(j\right)}$ reaches its minimum, that is, when the incremental intensity gain per unit exposure time is minimized. Discrete sampling and numerical difference method are implemented to solve this differential term, and obtain the optimal exposure time. At this optimal exposure time $t_{opt}^{\left(j\right)}$, acquired image data is effectively clustered in the temporal domain, separating valid intensity responses from saturated artifacts.

**Figure 4:**
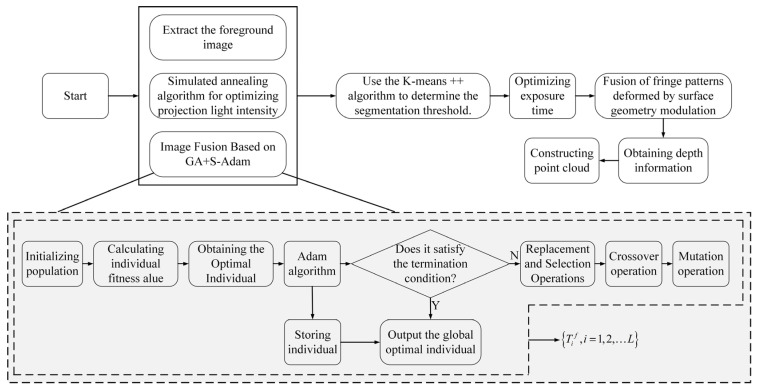
The main framework of the proposed method in this paper.

The sequence images corresponding to the optimal projection intensity are fused to generate a composite image. By analyzing the histogram of the foreground image, regions with different reflectivities are clustered using the K-means algorithm. The fused fringe patterns, which have been distorted due to modulation by mechanical components under different exposure times, are subsequently used for phase-based 3D reconstruction (PMP). Depth information is then computed to ultimately obtain the point cloud data of the measured workpiece. The main framework of the proposed method in this paper is illustrated in Figure 4.

The steps described in Figure 4 are detailed as follows:

Step 1: As described in Section 3, the optimal projection intensities are obtained using the simulated annealing algorithm. To ensure synchronization, the built-in GPIO interface of the camera is employed to receive external trigger signals from the projector, enabling communication between the camera and the projector.

Step 2: The optimal projection intensity sequence images from Step 1 are fused using the GA+S-Adam algorithm, as detailed in Section 4, to facilitate foreground image acquisition.

Step 3: The fused images from Step 2 are processed using the foreground extraction method proposed in Section 4 to isolate the foreground regions for further analysis.

Step 4: The histogram of the extracted foreground image is clustered using the K-means++ algorithm to determine segmentation thresholds, as illustrated in Section 4.

Step 5: The exposure time is determined based on the method introduced in Section 4, and the camera’s exposure settings are subsequently adjusted via computer control.

Step 6: Fringe images, modulated and deformed by mechanical components under varying exposure times, are fused to recover phase information.

Step 7: Finally, the 3D point cloud is reconstructed based on the system calibration parameters and the depth information obtained from decoding the fringe images.

When employing the simulated annealing algorithm to obtain the optimal projection intensity and acquire corresponding images via the camera, the determination of subsequent optimal intensities requires iterative computation. Once two or more images are obtained, image fusion under their respective optimal projection intensities can be performed in parallel. Given the continuous optimization of projection intensity and image fusion, foreground extraction from previously fused images can also be executed concurrently. The extracted foreground images are compared with the final fused image to guide the generation of foreground-specific histograms. These three stages can be processed in a pipelined manner to handle different batches of data, maximizing efficiency and reducing processing time.

## 5. Case Study

**Figure 5:**
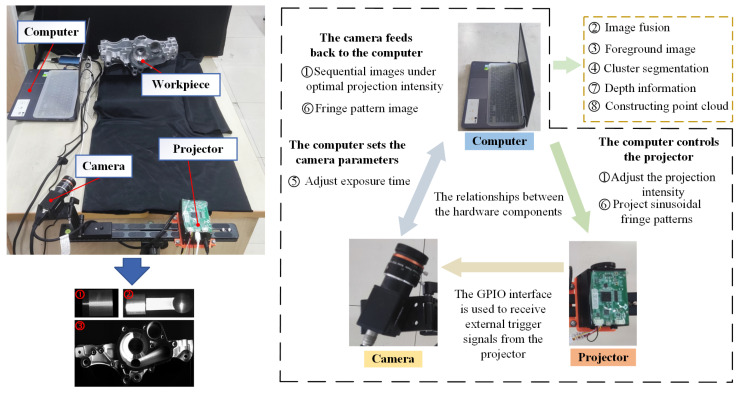
Configuration of the 3D reconstruction system.

A PMP-based 3D reconstruction system was constructed to validate the effectiveness of the proposed method, whose configuration is shown in Figure 5. This system comprises an MV-CA060-10GC CCD camera with a resolution of 3072 × 2048 pixels, a TJ-23U DLP projector with a resolution of 1280 × 720 pixels, and a high-performance computer. A heterodyne multi-frequency four-step phase-shifting method was employed for phase demodulation. The system software architecture consists of the following main modules: Projection Control Module: Responsible for generating and controlling ideal sinusoidal fringe patterns, which are projected onto the surface of mechanical components through the projector. Image Acquisition Module: Communicates with the camera through custom software to synchronize image acquisition. Data Processing Module: Integrates algorithms for projection intensity optimization, image fusion, foreground extraction, K-means++ clustering, optimal exposure time determination, system calibration, and 3D point cloud construction. Optimization algorithms are encapsulated as independent submodules to enhance maintainability. User Interface Module: Provides a visual interface for operation, supporting parameter configuration, real-time preview, and result output. To facilitate efficient collaboration between the projector, camera, and computing platform, a communication protocol is established. Projector to Host Communication: A USB 3.0 interface is used, with the projector’s dedicated SDK enabling real-time transmission and control of the projection patterns. Camera to Host Communication: The camera communicates with the host via the GigE Vision protocol, supporting high-bandwidth image data streaming. To ensure synchronization, the camera’s GPIO interface is employed to receive the external trigger signal from the projector, establishing communication between the camera and projector.

Three sets of experiments were conducted. The first group employed a geometrically uniform workpiece with highly reflective surface properties. The second group utilized a component featuring common mechanical geometries, including planar, cylindrical, and spherical surfaces, to validate the robustness of the proposed method across diverse reflective surface topologies. A die-cast automotive component was employed in the third group, in which there were multiple precision-manufactured end surfaces with different depths. Details and results of the three sets of experiments are shown as follows.

(1) Planar metal workpiece

**Figure 6:**
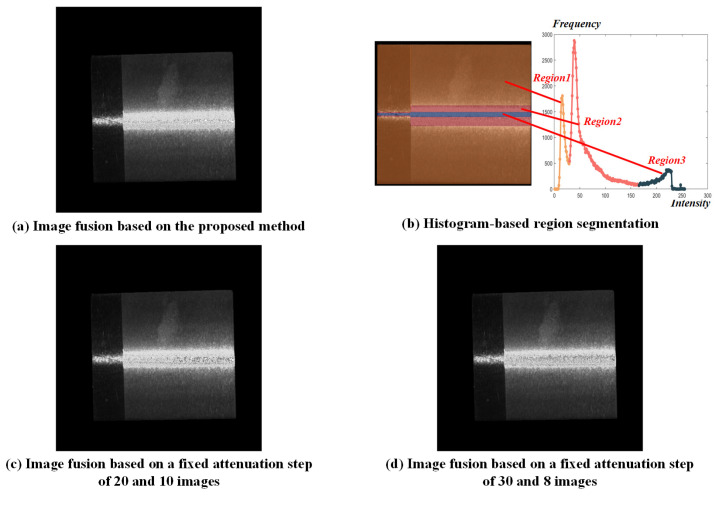
Image fusion based on the proposed methods and two conventional methods.

A planar metal workpiece, which is simple in geometry, was selected as the first example. The simulated annealing algorithm was employed to obtain the optimal projection intensities. The initial temperature was 100, the cooling coefficient was 0.95, and the termination temperature was 0.01. After convergence, the optimal projection intensities were 255, 214, 177, 152, 130, 113, 98, and 76, respectively. Image fusion was subsequently conducted based on the image sequence under the optimal projection intensity. The segmentation threshold was determined using a GA-ADAM algorithm, in which the population size was set to 20, the individual length to 8, and the initial crossover and mutation probabilities were set to 0.6 and 0.8, respectively. The hyperparameters for the ADAM algorithm were configured as $\alpha =1,\;\beta_{1}=0.9,\;\beta_{2}=0.999$. After convergence, a fused image was constructed based on the obtained segmentation threshold, as shown in Figure 6a. Based on the histogram-based analysis method, the pixels were clustered to three segmented regions as shown in Figure 6b, whose optimal exposure time were determined to be 35,264 μs, 5605 μs, and 3664 μs.

For comparison, two sets of conventional methods with fixed attenuation steps were employed. As the most frequently used step in the literature, the projection intensity attenuation step was set to 20 at first with a total of 10 fusion images, resulting in an optimal exposure time of 35,264 μs, 5129 μs and 3664 μs. In the second set, the attenuation step was set to 30 to ensure the same number of fused images with the proposed method, whose optimal exposure time were 35,264 μs, 5391 μs, and 3664 μs. Similarly, fused images were constructed after optimizing the segmentation thresholds using the GA-ADAM algorithm, as shown in Figure 6c and Figure 6d, respectively.

**Figure 7:**
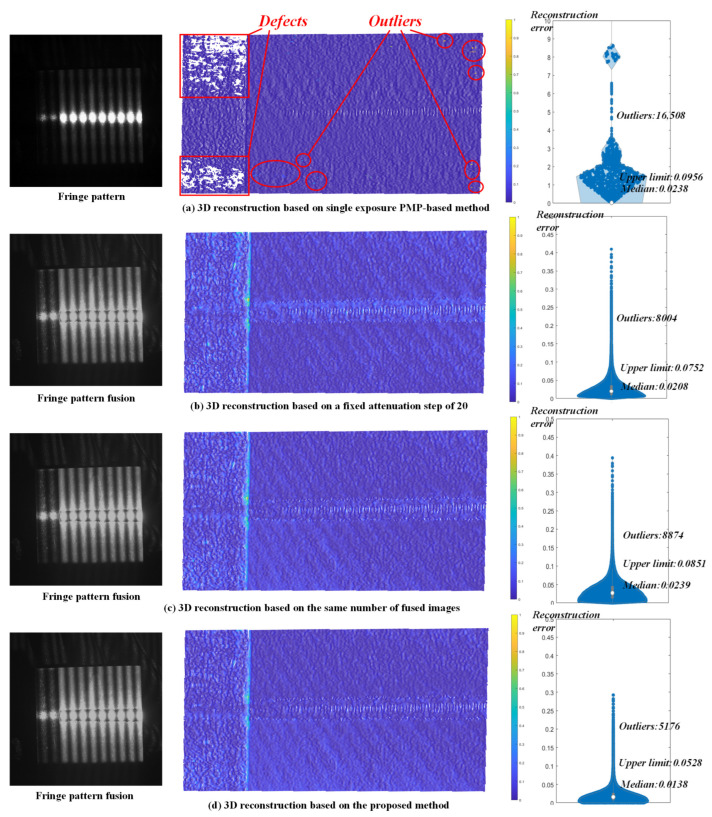
Comparison of 3D reconstruction results in case study 1.

Three-dimensional reconstruction was then carried out using the conventional PMP method, two sets of fixed attenuation step methods, and the proposed method. A single exposure with the maximum exposure time that just avoids image saturation was employed when using the conventional PMP method, while the obtained optimal exposure time were implemented in the other three scenarios. Three sets of sinusoidal fringes were projected onto the metal part. Each fringe comprised four patterns with different phase shifts, and the fringe periods were set to 90, 99, and 100, respectively. After capturing the projected fringes, the phases were unwrapped using a heterodyne multi-frequency phase-shifting method. The corresponding spatial data points were then localized based on the triangulation principle [45]. The workpiece was thus reconstructed by acquiring images at various exposure times and performing phase fusion. The results of 3D reconstruction based on the conventional PMP method, fixed attenuation steps of 20 and 30, and the proposed method are shown in Figure 7.

Reconstruction accuracy for each spatial point was calculated and statistically analyzed based on least squares planar fitting. A reference plane is fitted to the point cloud using the least squares method, resulting in a plane equation of the form ax+by+cz=0. For a given point $P\left(x_{p},y_{p},z_{p}\right)$, the distance from the point to the plane is calculated as: $d=\left|ax_{p}+by_{p}+cz_{p}+d\right|/\sqrt{a^{2}+b^{2}+c^{2}}$. The conventional PMP method which employs a single exposure for the entire region successfully avoided saturation but struggled to enhance the SNR in dark areas, which resulted in a 68.6% more in the number of outliers, 44.8% higher in the whisker range of reconstruction error, and 42.0% higher in the median errors comparing to the proposed method. In contrast, three methods based on image fusion produced reconstruction results with almost no geometric defects and achieved significantly lower reconstruction errors. The whisker range of the reconstruction error boxplot for the proposed method is 29.8% and 37.9% lower than that of the 20-step and 30-step methods, respectively, while the median errors are reduced by 33.7% and 42.3%, respectively. The whisker range is computed as: $max(x_{i}|x_{i}\leq Q_{3}+1.5\times IQR)-min(x_{i}|x_{i}\geq Q_{1}-1.5\times IQR)$, where $Q_{1}$ and $Q_{3}$ are the 25th and 75th percentiles, and the interquartile range is defined as $IQR=Q_{3}-Q_{1}$. These results demonstrate the effectiveness of the proposed method and its higher reconstruction accuracy compared with commonly used existing methods.

(2) Multi-geometry metal workpiece

**Figure 8:**
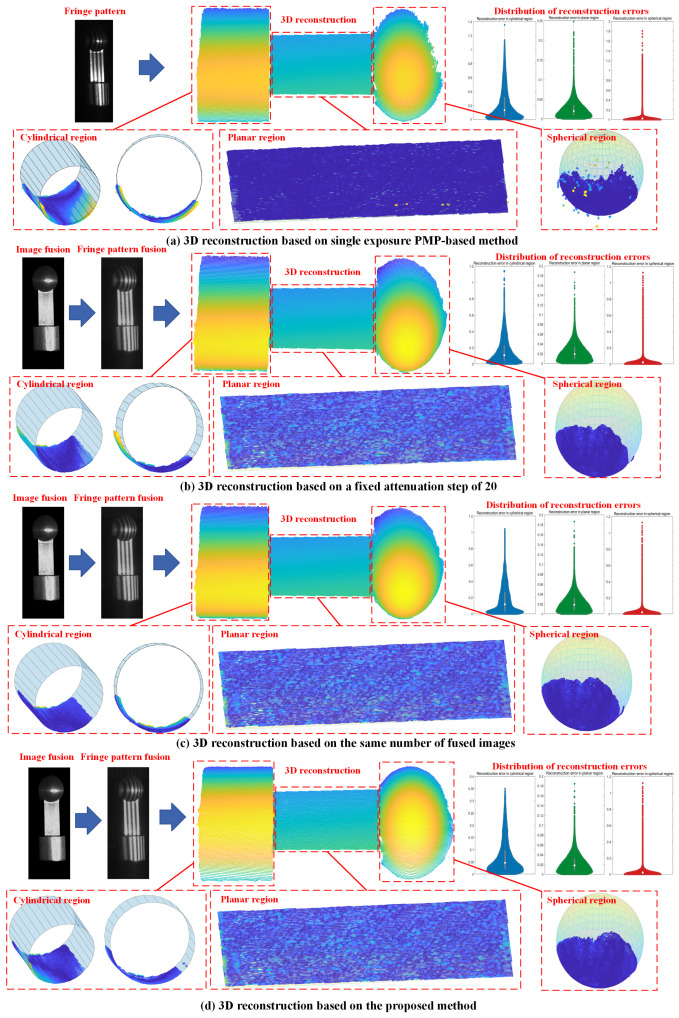
Comparison of 3D reconstruction results in case study 2.

Further, a metal component with a slightly more complex geometry, featuring typical mechanical characteristics such as planar surfaces, through-holes, outer cylindrical surfaces, and spherical surfaces, was selected as the second example. Image fusion was conducted based on nine images, which were under the obtained optimal projection intensity: 255, 210, 172, 147, 128, 110, 89, 73, and 58. Three clusters of pixels were segmented based on the proposed method, and the corresponding optimal exposure time were 9159 μs, 18,398 μs, and 32,058 μs. In the conventional PMP-based 3D reconstruction, a single exposure with the maximum exposure time was used to increase the SNR. When using the methods based on fixed attenuation steps, a step size of 25 was employed to ensure that the fixed attenuation step method and the proposed method could use the same number of fused images. The conventional step size of 20 was also used for the comparison experiment. The obtained optimal exposure time were 9159 μs, 14,150 μs, 32,058 μs; 9159 μs, 15,444 μs, 32,058 μs, respectively for steps of 20 and 25. The results of 3D reconstruction are shown in Figure 8.

Reconstruction errors were quantified by segmenting local point clouds and performing least-squares fitting to cylindrical, planar, and spherical geometries. When fitting a cylindrical surface, the axis parameters include the direction vector $\vec{D}=(d_{x},d_{y},d_{z})$, a point on the axis $P_{0}\left(x_{0},y_{0},z_{0}\right)$, and the radius R. Given a point cloud $\left\{P_{i}=(x_{i},y_{i},z_{i})|i=1,2,\ldots ,N\right\}$, the parameters are estimated by minimizing the objective function: $J=\sum_{i=1}^{N}{\left(\sqrt{(x_{i}-x_{0}-t_{i}d_{x}{)}^{2}+(y_{i}-y_{0}-t_{i}d_{y}{)}^{2}+(z_{i}-z_{0}-t_{i}d_{z}{)}^{2}}-R\right)}^{2}$, the parameters $x_{0},y_{0},z_{0},d_{x},d_{y},d_{z},R$ are obtained, where $t_{i}=(x_{i}-x_{0})d_{x}+(y_{i}-y_{0})d_{y}+(z_{i}-z_{0})d_{z}$. For spherical surface fitting, the least squares method is used to estimate the sphere center $C=\left(x_{c},y_{c},z_{c}\right)$, and radius R. Given a point cloud $\left\{P_{i}=(x_{i},y_{i},z_{i})|i=1,2,\ldots ,N\right\}$, the objective function is defined as: $J=\sum_{i=1}^{N}{\left(\sqrt{(x_{i}-x_{c}{)}^{2}+(y_{i}-y_{c}{)}^{2}+(z_{i}{-z}_{c}{)}^{2}}-R\right)}^{2}$, the parameters $x_{c},y_{c},z_{c},R$ are obtained. Comparison of statistics of reconstruction errors in three regions is listed in Table 1. Compared with conventional PMP-based methods, the proposed method reduced the whisker range by 65.2%, 50.9%, and 47.4% for cylindrical, planar, and spherical regions, respectively, while reducing the median error by 63.6%, 13.0%, and 24.7%. When compared with the image fusion method with a fixed attenuation step of 20 which is commonly employed in existing studies, the proposed method showed a reduction in error ranges by 52.7%, 4.3%, and 0.3%, and a reduction in median errors by 33.9%, 8.7%, and 7.3% respectively across the three regions. In comparison with the image fusion method with a fixed attenuation step of 25 to ensure the same number of fused images, the error range is reduced by 57.4%, 1.2%, and 0.3%, and the median error is reduced by 50.6%, 5.4%, and 8.5%. These results validate the superior reconstruction accuracy of the proposed methodology.

**Figure 9:**
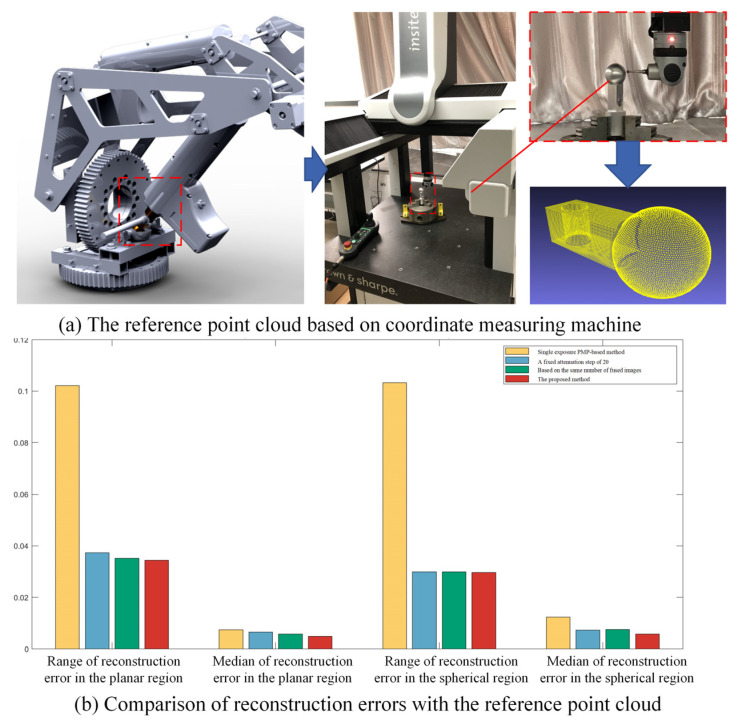
The reference point cloud based on coordinate measuring machine.

A reference point cloud was obtained using a coordinate measuring machine (CMM) to mitigate the influence of machining-induced surface uncertainties in the fitting-based estimation of reconstruction error. Then, the reconstruction errors were benchmarked against the CMM-measured form errors. Because the cylindrical surface was clamped by a three-jaw chuck on the workbench, programmed CMM scanning was only feasible for the upper spherical and planar regions to obtain the reference point cloud. The measurement setup and results are illustrated in Figure 9a. Owing to the finite radius of the CMM’s ruby-tipped probe, narrow crevices at the spherical-planar intersections remained unmeasurable, resulting in some holes in the corresponding area. After processing the reference point cloud, the range and median of form error for the planar region are 0.03087 and 0.01265, while the range and median of form error for the spherical region are 0.05183 and 0.01437, respectively. In consideration of the superior accuracy of CMM over that of vision-based measurement methods, these data are considered as ground truth. As shown in Figure 9b, the proposed method achieved an average reduction of 25.5% in the whisker range and 25.9% in the median of reconstruction error for planar regions compared to three existing methods. For spherical regions, the average reductions reached 24.2% in error range and 33.1% in median error.

(3) High-precision aluminum die-casting component

A precision aluminum die-casting workpiece for new energy vehicles was selected as the third validation case. The die-cast component contains multiple machined hole end faces with different depths, which were machined by precision milling and grinding processes. As these surfaces have passed quality inspection using CMMs with an accuracy of 0.1 μm, they exhibit high-dimensional accuracy, indicating their potential in serving as benchmarks for evaluating the accuracy between vision-based non-contact 3D reconstruction methods.

**Figure 10:**
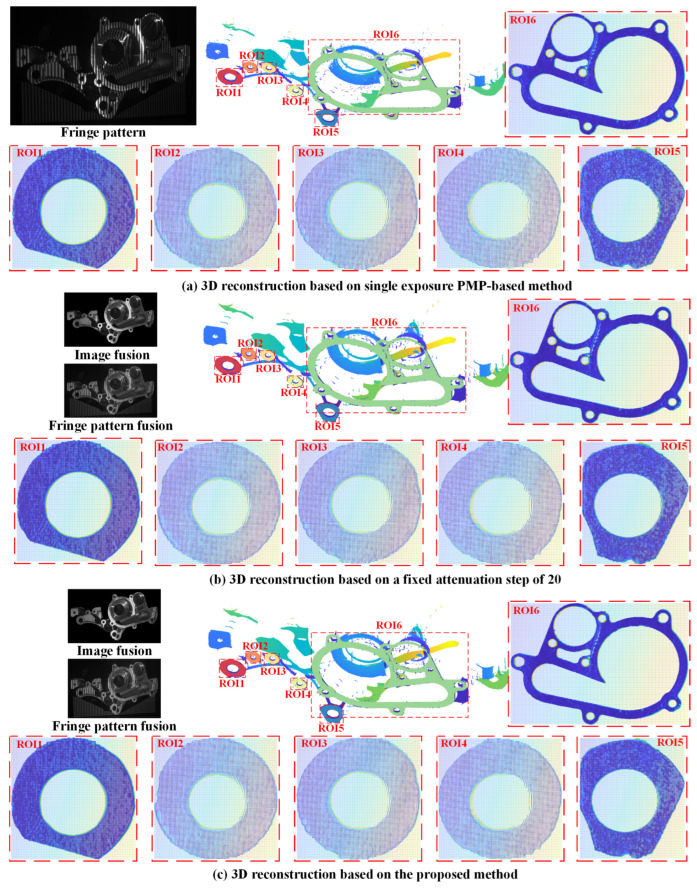
Comparison of 3D reconstruction results in case study 3.

Using the proposed method, image fusion was conducted based on twelve images, which were under the obtained optimal projection intensity: 255, 216, 184, 155, 131, 110, 92, 76, 63, 51, 45, and 26. The optimal exposure time were 12,823 μs, 17,222 μs, and 31,851 μs. Similarly, the exposure time were maximized in the conventional PMP-based method to increase SNR and reconstruction accuracy. The conventional step size of 20 was used for comparison, in which the obtained optimal exposure time were 12,823 μs, 16,187 μs, and 31,851 μs. The results of 3D reconstruction are presented in Figure 10.

**Figure 11:**
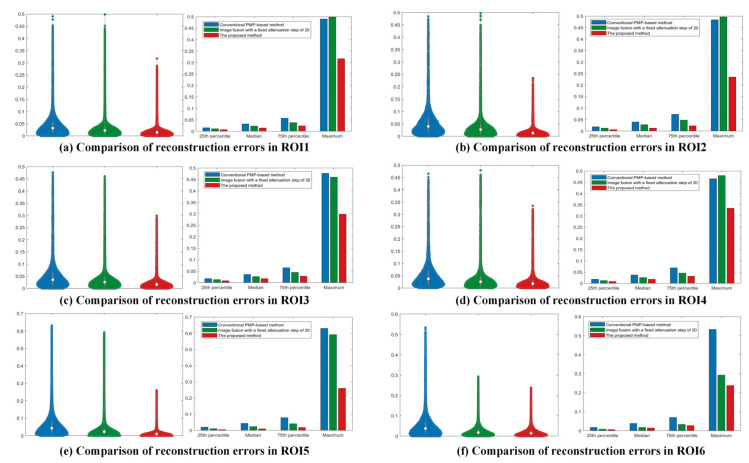
Comparison of reconstruction error in case study 3.

Six precision-machined hole end faces with varying depths were segmented using a random-seed region growing algorithm, and reconstruction errors in six ROIs were estimated through least-squares plane fitting. Compared to the conventional PMP-based method, the proposed method achieved average reductions of 63.6%, 64.1%, 65.6%, and 47.6% in the 25th percentile, median, 75th percentile, and whisker range of reconstruction errors, respectively. The 25th and 75th percentiles are defined as: given a sorted dataset $x_{1},x_{2},\ldots x_{n}$, the position of the percentile is calculated by: $i=1+\left(n-1\right)\times p$, where p is the decimal form of the desired percentile (e.g., p = 0.25 for the 25th percentile and p = 0.75 for the 75th percentile). Compared to an image fusion method with a fixed step of 20, the proposed method reduced the reconstruction error by 44.6%, 37.1%, 45.8%, and 41.3% on average, respectively. The experimental results empirically validate the superior reconstruction accuracy of the proposed methodology. The comparison of reconstruction error is shown in Figure 11.

The efficiency of different reconstruction methods for highly reflective metallic components is intuitively illustrated through a statistical analysis of data acquisition and processing cycles. The time-consuming steps primarily include obtaining the optimal projection intensity and corresponding mechanical part images, image fusion, threshold segmentation, and result extraction. It is worth noting that factors such as the algorithm, camera, projector, computing hardware, and the complexity of the workpiece (e.g., geometric complexity and reflective properties) can significantly affect the processing time. A detailed analysis of the aforementioned steps is provided below:

(1) Acquisition of Projection Intensity and Corresponding Mechanical Part Images.

For the three representative cases, the time required to determine a single optimal projection intensity was approximately 2 s, 2 s, and 3 s, respectively. Additionally, the process of determining projection intensity using a fixed-step attenuation strategy, followed by synchronous image acquisition with the camera, took approximately 4 s per cycle.

(2) Image Fusion.

In the three representative cases, the time required for image fusion was approximately 8 s, 9 s, and 12 s, respectively.

(3) Threshold Segmentation.

Threshold segmentation is performed using the K-means++ algorithm. The processing time required for this step was approximately 4 s, 4 s, and 5 s for the three respective cases.

(4) Result Acquisition.

Determining the optimal exposure time takes around 2 s. Capturing a fringe image under a single exposure condition takes approximately 3 s. The phase fusion of fringe images with the same phase shift within the same exposure cycle takes approximately 4 s, 4 s, and 5 s for the three representative cases. Finally, reconstructing the 3D point cloud from the calibration parameters and depth information requires approximately 3 s, 3 s, and 4 s, respectively.

The efficiency of different methods for reconstructing highly reflective metallic components is intuitively demonstrated by statistically analyzing the required processing time. In case 1, the total durations required by the conventional PMP method, the fixed attenuation step method, the proposed method integrated with a consistent attenuation pattern, and the proposed method alone are 6 s, 43 s, 42 s, and 54 s, respectively. In case 2, the corresponding total durations are 6 s, 44 s, 43 s, and 57 s. In case 3, the total durations required by the conventional PMP method, the fixed attenuation step method, and the proposed method are 7 s, 51 s, and 83 s.

From the above analysis, it is evident that the conventional PMP method exhibits the highest efficiency but the lowest reconstruction accuracy. Although the proposed method requires a longer processing time compared to other approaches, it achieves the highest reconstruction accuracy. The fixed-step attenuation method, on the other hand, offers a balance, with processing time and accuracy falling between the conventional PMP method and the proposed method. Therefore, in future applications, a reconstruction method can be selected based on a reasonable trade-off between efficiency and accuracy.

## 6. Conclusions

As for the lack of effective guidance in the imaging parameters in PMP-based 3D reconstruction, an adaptive high-precision 3D reconstruction of highly reflective mechanical parts based on optimization of projection intensity and exposure time is proposed in this paper. An image sequence is established during the search for optimal projection intensity to achieve the best hardware performance. A GA-SAdam framework is also proposed to maximize the retained details in the image fusion process. The exposure time is adaptively adjusted based on the surface reflective properties.

Three sets of typical mechanical parts were conducted in the case study, which comprises varying geometric shapes and reflective characteristics. Experiment results show that compared with the existing single-exposure method, a commonly-used attenuation step method, and a fixed-step based method with the same number of fused images, the proposed method reduced the average whisker range of reconstruction error by 51.18%, 25.68%, and 24.20%, and decreases the median error by 41.48%, 24.14%, and 26.70%, respectively. The effectiveness and high accuracy performance of the proposed method have been verified.

The currently adopted projector and camera setup may be inadequate for covering larger-scale components. This limitation can be addressed through hardware upgrades to accommodate a wider field of view for both projection and imaging. Enhancing the projector’s output power can mitigate the attenuation of projection intensity over large areas, which otherwise degrades fringe quality, particularly on highly reflective surfaces. Real-time inspection demands lightweight algorithms (e.g., compressed deep learning) and hardware optimizations (e.g., FPGAs) to meet latency constraints. Trade-offs between accuracy and speed must be balanced, but hybrid approaches (e.g., adaptive fusion + edge computing) could enable robust deployment in industrial settings.

It is worth noting that the proposed methodology minimized manual intervention through the integration of programmable fringe projectors, customized camera software development, optimization algorithms, and coordinated communication protocols. However, the required number of experiments escalates exponentially with increasing segmented regions and iteration steps. Furthermore, a substantial portion of experimentally acquired images contains underutilized regions, which indicates that correlation analysis of imaging parameters could be focused on to enhance data utilization and searching efficiency. It is worthwhile to investigate a better experimental design to promote real-time performance and practical deployment in industrial scenarios.

## Figures and Tables

**Table 1:** Comparison of statistics of reconstruction errors in three regions between the single-exposure PMP-based method, image fusion based on a commonly used fixed attenuation step, and the same number of fused images.

Region	Statistical Feature	Single-Exposure PMP-Based Method	Image Fusion with a Fixed Attenuation Step of 20	Image Fusion with the Same Number of Fused Images	The Proposed Method
Cylindrical region	Spread range	0.7387	0.5443	0.6032	0.2572
	Median error	0.1788	0.09855	0.1320	0.06517
Planar region	Spread range	0.1330	0.06818	0.06605	0.06525
	Median error	0.02010	0.01916	0.01848	0.01749
Spherical region	Spread range	0.1551	0.08174	0.08173	0.08152
	Median error	0.02673	0.02170	0.02199	0.02012

## Data Availability

The data in this study are available upon request from the corresponding author. The data are not publicly available due to privacy.
